# Resilience to climate change by improving air circulation efficiency and pollutant dispersion in cities: A 3D-UFO approach to urban block design

**DOI:** 10.1016/j.heliyon.2024.e36904

**Published:** 2024-08-30

**Authors:** Mehdi Makvandi, Philip F. Yuan, Qunfeng Ji, Chuancheng Li, Mohamed Elsadek, Wenjing Li, Ahmad Hassan, Yu Li

**Affiliations:** a College of Architecture and Urban Planning, Tongji University, Shanghai, China; b College of Civil Engineering and Architecture, Wuhan University of Technology, Wuhan, China; c College of Architecture and Urban Planning, Huazhong University of Science and Technology, Wuhan, China; d Key Laboratory of Ecology and Energy-saving Study of Dense Habitat, Ministry of Education, Shanghai, China

**Keywords:** Urbanization, Land-use and land-cover change (LULCC), Air circulation efficiency, CFD, 3D urban form optimization (3D-UFO)

## Abstract

Urbanization presents significant challenges to air quality and climate resilience, necessitating pioneering urban design solutions to enhance air circulation and mitigate pollutants. This urgency intensifies in densely populated and rapidly evolving regions like Wuhan, China, where effective strategies are crucial for sustainable development. This study introduces an innovative 3D Urban Form Optimization (3D-UFO) methodology aimed at advancing urban block design configurations to improve urbanization quality. The 3D-UFO approach systematically addresses the multifaceted challenges of climate change and air quality degradation in rapidly urbanizing areas. Integrating GIS-based analysis for comprehensive Land-Use and Land-Cover Change (LULCC) evaluation with Computational Fluid Dynamics (CFD), our approach employs systematic exploration guided by established urban airflow study protocols. Robust metrics—Airspeed-Ratio (ASR) and Average-Age-of-Local-Air (ALA)—quantify the impact of diverse urban block design strategies on air-circulation efficiency and pollutant dispersion. Analysis across various urban scenarios, yielded by the proposed 3D-UFO approach, reveal significant variations in air-circulation efficiency at street and building levels (SBLs). Optimal urban air circulation achieves efficiency levels of 50–70 % when airflow aligns orthogonally across and parallel to streets. Adjusting street-level building heights, especially incorporating taller structures, boosts ventilation efficiency by 20–30 %, which is crucial for improving airflow dynamics in urban settings. Higher Height-to-Width (H/W) ratios (>5.5) yield a 218.5 % increase in ventilation in specific urban layouts. Notably, the synergy of street-aspect-ratio and building-height-ratio adjustments significantly enhance ASR and ALA, providing a quantitative foundation for sustainable urban development. This 3D-UFO methodology, fusing LULCC analysis, CFD simulations, and systematic exploration, emerge as a valuable framework for urban planners and designers. The study offers informed insights into urban sustainability challenges, demonstrating advancements in addressing environmental concerns and improving living conditions within densely populated environments.

## 1 Introduction

Urbanization, driven by population growth and land use changes, profoundly affects socio-economic development and local climate dynamics, affecting wind patterns, air quality, and human comfort [1,2]. In China, this phenomenon exhibited a notable increase from 17.9 % in 1978 to 59.6 % in 2018, necessitating sustainability efforts to mitigate its impact on land use, urban structure, and the local temperature regimes [[3], [4], [5]].

**Fig. 1:**
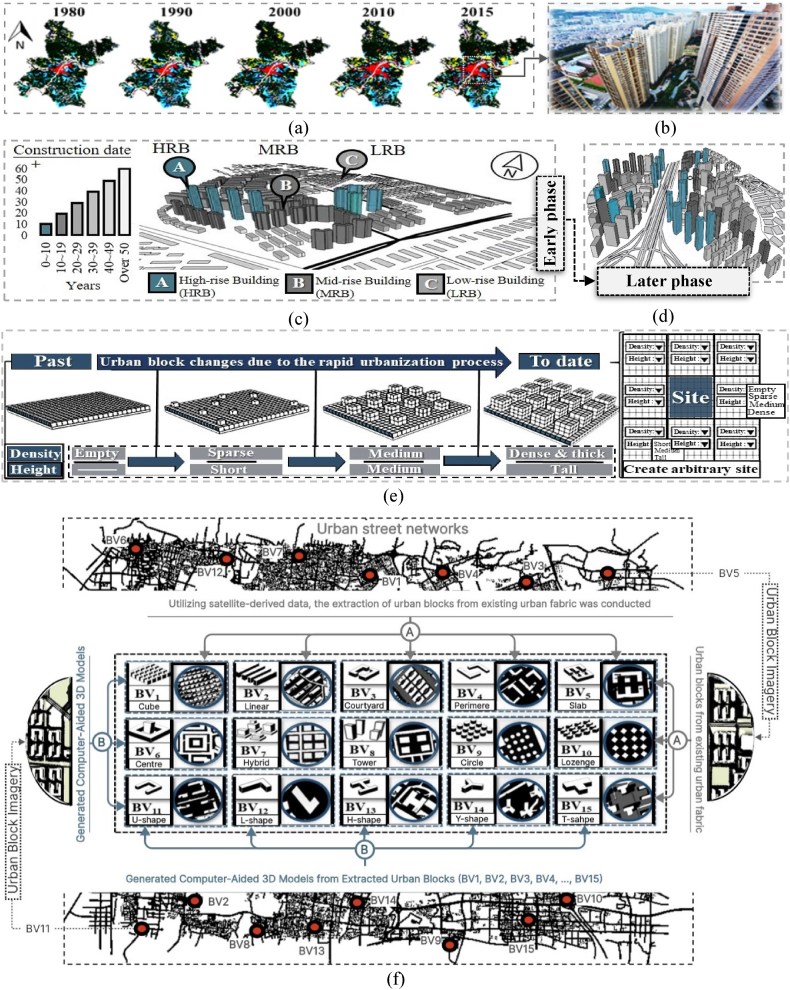
Wuhan's urban development overview: (a) Built-up areas highlighted in red; (b) Urban view from a residential peak; (c) Early urban development and (d) later urban development phases (south bird-eye-view); (e) Evolution of typical building forms under urbanization regulations; (f) Samples of urban blocks across fifteen typology categories.

In rapidly urbanizing cities like Wuhan, with an annual development rate of 1.12 %, challenges emerge due to prioritizing expansion over comprehensive planning [6]. This leads to densely populated regions facing air quality and heat-related challenges, exacerbated by imbalances in urbanization speed and quality, despite recent government regulations (Fig. 1e) [[7], [8], [9]]. In urban environments, building blocks' configuration and morphology are key factors for shaping thermal dynamics and mitigating challenges related to land-use and land-cover changes, including heat stress, airflow disruptions, and pollutant concentrations [[10], [11], [12]].

The objective of this study is to address these urbanization challenges by optimizing urban blocks for better urbanization quality using an innovative 3D Urban Form Optimization (3D-UFO) approach. Validated computational fluid dynamics (CFD) simulations to investigate and improve air circulation in densely built-up areas like Wuhan enrich this approach.

CFD serves as a cost-effective method for assessing urban forms' influence on natural ventilation [13,14]. Nonetheless, it faces limitations, particularly in turbulence modeling accuracy under real-world conditions [15]. Fig. S1 highlights limitations in existing research methods, from model acquisition and generation to CFD simulation. Analysts have scrutinized different turbulence models based on Reynolds-averaged Navier-Stokes (RANS) for accuracy, with each addressing specific ventilation challenges [[16], [17], [18], [19], [20], [21]].

To enhance simulation precision, researchers have systematically studied factors such as computational domain size, grid resolution, atmospheric boundary layer profiles, discretization scheme order, and convergence criteria [22]. The standard k-ε model has demonstrated superior ventilation performance in generic building scenarios, aligning with experimental observations [23]. Moreover, various modeling methods have been proposed for CFD simulations in urban environments [[24], [25], [26], [27]].

In urban ventilation studies, objectives can be categorized based on (1) the creation of 3D forms and (2) their complexities. The first category primarily uses simple forms, such as solitary cubic-shaped blocks [28,29], and urban canyon geometries [30,31] to examine building aspect ratio [32], street aspect ratio (H/W) [33], building density (BD) [34], building height variations (BHV) [35] and their impact on airflow and pollutant dispersion [36,37]. The second category investigates realistic 3D urban spaces for more sophisticated evaluations of outdoor/indoor ventilation within diverse urban contexts, including real compact heterogeneous areas [38], multi-story structures [39], canyons and pedestrian levels [40].

As evident in the literature, previous studies on urban ventilation conditions and thermal comfort in model-based urban configurations [41] have often overlooked critical factors like design ideologies, local adjustments, and socio-economic influences within urban blocks. The core objective of this study is to bridge the gap between the predominant simplistic cuboidal geometries found in urban design literature and the complex dynamics present in real-world urban environments, utilizing the 3D-UFO approach across diverse site scenarios. This holistic approach provides a comprehensive examination and evaluation of air quality concerns in rapidly evolving urban areas, considering historical development trends, regulatory frameworks, urban design parameters, idealized urban configurations, site-specific features, and ventilation performance assessment. A key study feature is incorporating block design scenarios, highlighting CFD models' pivotal role in environmental dispersion research.

Through a detailed analysis of urban development, building block diversity, and critical ventilation metrics like Average-Age-of-Local-Air (ALA) and Airspeed Ratio (ASR), our approach addresses disparities, providing a comprehensive understanding of air quality concerns and contributing to optimizing urban air circulation efficiency.

The proposed 3D-UFO approach investigates urban air quality in Wuhan's evolving urban areas through four facets: (1) the city's historical growth, urban planning, and building regulations; (2) extract the vital urban design parameters; (3) creation of idealized urban forms characterizing optimal land distribution within a generic street network; and (4) the ventilation performance of these forms using validated CFD simulations. This approach presents an effective 3D urban design solution, providing valuable insights for city stakeholders to implement.

## 2 3D-UFO creation for urban block evolution

Antecedent to CFD analysis, we extensively applied remote sensing and Geographic Information System (GIS) techniques to investigate the region's land-use and land-cover changes (LULCCs). This encompassed an analysis of the historical development of urban building blocks across diverse sites from 1980 to 2015. After analyzing hundreds of urban blocks, we developed practical 3D urban design frameworks considering (1) various physical characteristics (e.g., H/W, BHV, and BD), site-configuration, and block types, (2) urban design principles, (3) building block regulations, and (4) developers' profit-driven motivations.

Subsequently, we created and assessed groups of 3D urban forms using well established CFD models and validated assessment indices like mean air age and air change rate. Our innovative 3D-UFO approach enables the generation of urban block configurations compliant with planning regulations while enhancing ventilation efficiency, evaluated through the ASR and ALA indices at street and building levels (SBLs).

### 2.1 Wuhan's urban development

Wuhan in central China (113°41′-115°05′E, 29°58′-31°22′N) covers 8569.15 km^2^, featuring plains amid mountains [42]. Urban land, once less than 9 % (759.5 km^2^) in 1980, expanded to nearly 36 %, driven by rapid population growth from 2.52 million to 11.08 million over 40 years, causing a 405 % increase in urban areas and decreasing water bodies to 1.17 % (Fig. 1a) [42,43].

This shift impacted the wind environment, cooling demands, and land surface temperatures, intensifying the urban heat island effect. To accommodate the population surge, Wuhan's urban planning regulations evolved, leading to diverse urban block generations and building forms as shown in Fig. 1e. Wuhan's building and planning regulations underwent revisions to accommodate this population growth, leading to changes in urban blocks and building forms, as illustrated in Fig. 1e and f, respectively. During each timeframe, developers maximized built spaces within the confines of regulatory provisions and endeavored to select building forms aligned with purchaser preferences.

This transformed Wuhan into a partially high-density, high-rise urban area [44], as revealed in Fig. 1b. Current regulations permit total site coverage for buildings under 20 m and impose plot ratio limits from 8 to 10. This led to cake-like low-rise, mid-rise, and tall buildings, progressively evolving Wuhan's urban form. In Fig. 1c, the Jiangxia area in Wuhan is still in the initial transformation phase, while Fig. 1d depicts a neighborhood in an advanced development stage. Over the past decade, many sites initially undergoing these transformations have been developed as high-rise structures. Wuhan is expected to ultimately become a total high-density, high-rise urban setting under current regulations, necessitating optimization of 3D urban forms through the 3D-UFO approach proposed here.

### 2.2 Critical variables in realizing 3D urban Form optimization (3D-UFO)

**Fig. 2:**
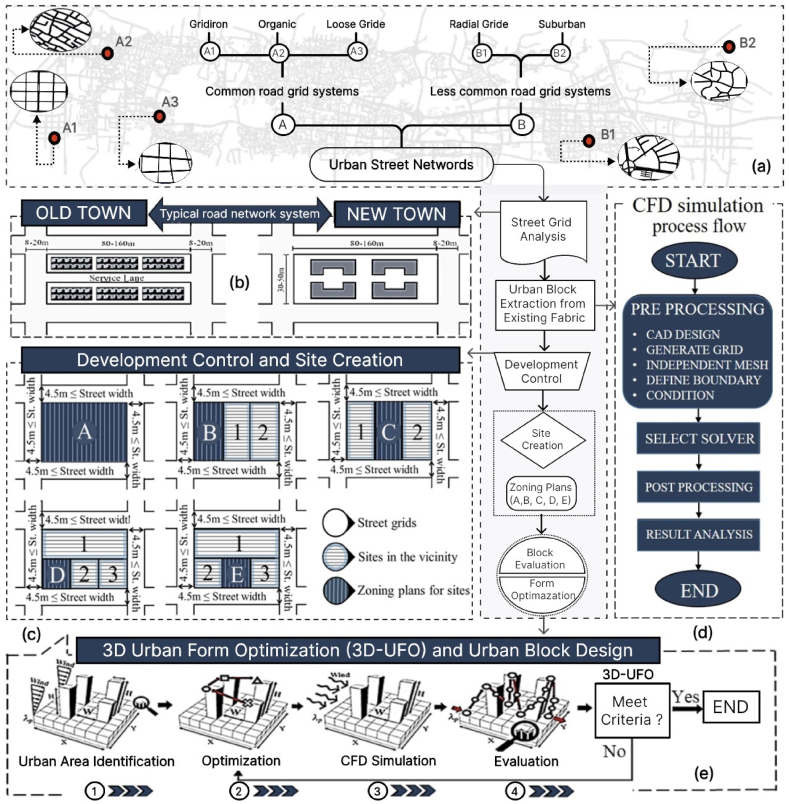
(a) Comparison of common and less common road network systems, delineating distinctions in layout and connectivity; (b) Typical road network system for residential areas within the Wuhan metropolitan area, highlighting street patterns and urban design; (c) Site variations in Wuhan influenced by Development Control Regulations (DCR), showing changes in building layouts and planning approaches.; (d) Detailed process flow of a CFD simulation, outlining the steps from model setup to result analysis; (e) Schematic diagram of the proposed framework, depicting the sequential steps used in the study.

In the quest for optimal urban development and environmental quality, certain morphological variables emerge as critical determinants. Urban road networks, site types, and building forms are pivotal elements in realizing 3D-UFO. Urban road networks hold a prominent role in urban design and overall cityscape configuration [45,46]. Extensive road network analysis in Wuhan reveals a duality in its urban fabric. The modern sector boasts a European-inspired road network featuring diverse layouts, including gridiron, loose grid, organic, radial grid, and suburban systems (Fig. 2a) [7,47,48]. Conversely, old-established areas predominantly feature an oblong road network, with street blocks exhibiting variations in width (30–50 m) and length (80–160 m), interspersed with road widths spanning 8–20 m (Fig. 2b). The presence of service roads further delineates distinctive urban forms in these areas. In the modern sector, while a comparable road network system is implemented, service routes are omitted due to the prevalence of high-rise building development.

Over a meticulous 35-year investigation into residential growth in Wuhan, we systematically classified sites into five groups, strictly adhering to Development Control Regulations (DCR) (Fig. 2c) (https://www.soujianzhu.cn/NormAndRules). These site classifications, comprising A, B, C, D, and E types, form the bedrock of 3D-UFO. Type-A sites are expansive areas enclosed by four roads wider than 3.5 m, boasting plot ratios ranging from 7.6 to 9.7. Type-B sites are adjacent to three roads wider than 3.5 m, while Type-C and Type-D sites each adjoin two such roads, with average plot ratios of 10.5, 8.4, and 9.4, respectively. Type-E sites are areas bordering one road wider than 3.5 m, with an average plot ratio of 8.4. According to Wuhan's regulations, plot ratios can increase by 0.1 times with the integration of specific green features into building design. Our investigation of residential growth unveiled a profound interplay between building parameters (H/W, BD, BHV) and site forms, significantly impacting air circulation efficiency and thermal environments. This intricate relationship is encapsulated within the framework of 'environmental optimization principles (EOP)' for 3D-UFO. In the densely populated urban context of Wuhan, each site category's outlook toward adjoining roads significantly impacts building characteristics' value. Developers and planners seek optimized designs aimed at enhancing environmental conditions. Fig. 2e concisely illustrates the site optimization procedure, encompassing urban area identification to 3D-UFO realization, complemented by Fig. 2d, which provides a comprehensive overview of the entire CFD process flow employed in the study's investigations.

To advance our understanding, Type-A sites are conducive to high-rise buildings, characterized by structures surrounding the core and a larger surface area. Building varieties (BV) such as cube (BV_1_), linear (BV_2_), courtyard (BV_3_), perimeter (BV_4_), slab (BV_5_), centralized (BV_6_), hybrid (BV_7_), tall building/tower (BV_8_), circular (BV_9_), and lozenge (BV_10_) are predominantly adopted within this category. In Type-B sites, the U-formed layout (BV_11_) is frequently use, connecting three adjacent streets and offering expansive vistas and open spaces. Type-C sites, featuring two parallel adjacent roads, often utilize an H-formed layout (BV_13_). Typically, Type-D sites employ L-formed structures (BV_12_) along the road, while Type-E sites along a single road prioritize configurations that optimize the width across the street for enhanced visibility and utilization of open spaces. Y-formed (BV_14_) and T-formed (BV_15_) configurations are also favored for Type-D and Type-E sites, respectively. These aspects, coupled with the scenarios delineated in Section 2.3, constitute the fundamental framework of the study's 3D-UFO approach, furthering our understanding of Wuhan's densely populated urban environment.

### 2.3 Developing design strategies for 3D-UFO and modeling

**Fig. 3:**
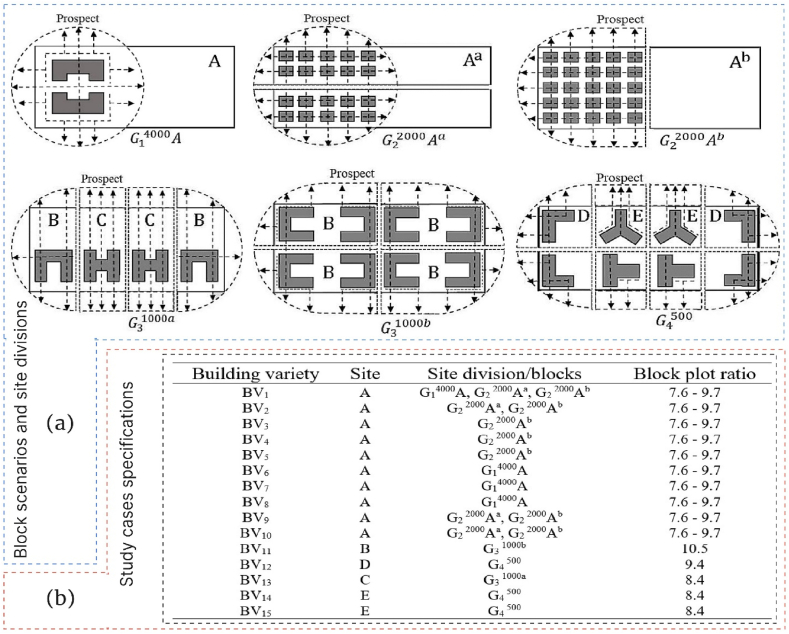
(a) Analyzing block scenarios and site divisions with common building types in 3D-UFO approach and (b) Specifications of study cases, detailing relevant parameters and configurations.
ii. Plot ratios: Within the framework of Wuhan's DCR, we meticulously imposed plot ratio limits for building construction and planning, with a deliberate focus on their impact on the surrounding environment. Our analytical approach drew upon Height-to-Width ratios (H/W) and Building Height Variation (BHV), yielding nuanced variations in plot ratios tailored to the specific site types. See Fig. 3b for detailed scenario descriptions. It is imperative to note that interactions between building forms and block arrangements yielded minor adjustments in plot ratios and other pertinent building parameters.
iii. Building Form in Block Configuration Scenarios: we meticulously selected building varieties (BV_1_ to BV_15_) with a primary emphasis on building forms. This precise selection process deepens our comprehension of the influence of building shape on urban design. Small G_4_^500^ blocks, encompassing D and E sites, pertain to buildings with diminutive footprints/floor areas like BV_12_, BV_14_, and BV_15_, while average-scale blocks (G_2_^2000^A^a^, G_3_^1000a^, and G_3_^1000b^) correlate with buildings featuring intermediate footprints/plans, exemplified by BV_11_ and BV_13_. Moreover, BV_10_, BV_9_, BV_2_, and BV_1_ may also fall into the medium category, provided they exhibit mid-density attributes (i.e. H/W = 1/1 and BD = 0.3). Spacious blocks like G_1_^4000^A, as well as average blocks with mid-density and low aspect ratios like G_2_^2000^A^b^, are associated with larger-scale building projects, exemplified by BV_8_, BV_7_, BV_6_, BV_5_, BV_4_, and BV_3_. Furthermore, extensive site plan adjustments and building parameter modifications allow for the analysis of BV_10_, BV_9_, BV_2_, and BV_1_ within this category.

In our pursuit of effective 3D Urban Form Optimization (3D-UFO) and the development of design strategies, we meticulously crafted six distinct scenarios that align seamlessly with current regulatory frameworks governing road networks, land parcel delineations, and socio-economic considerations under urbanization process. Our paramount objective has been to formulate design strategies for 3D-UFO and modeling, which encompass the following key aspects.

i. Functional zoning: To commence, we subdivided a typical urban street area into four distinct block sizes—namely, G_1_^4000^ (4000 m^2^), G_2_^2000^ (2000 m^2^), G_3_^1000^ (1000 m^2^), and G_4_^500^ (500 m^2^). 'G' identifies the group categories. Within this framework, we executed a dual zoning strategy, comprising two distinct approaches: (1) on-site development without service paths and (2) block partitioning with the inclusion of a service path to create smaller categorized sites. For instance, Site A (G_1_^4000^), denoted as G_1_^4000^A, encompassing a total area of 4000 m^2^, underwent subdivision through the introduction of a horizontal or vertical service lines, yielding distinct A^a^ or A^b^ blocks designated as G_2_^2000^A^a^ and G_2_^2000^A^b^, respectively. Consequently, this transformation gave rise to additional cases—namely G_3_^1000a^, G_3_^1000b^, and G_4_^500^. Following Wuhan's Building and Planning Regulations (BPR), which adhere to the Development Control Regulations (DCR), service paths were designed to facilitate both ingress and departure, aligning with Site A's layout. This meticulous effort resulted in the creation of six distinct functional zoning scenarios, as depicted in Fig. 3a.

**Fig. 4:**
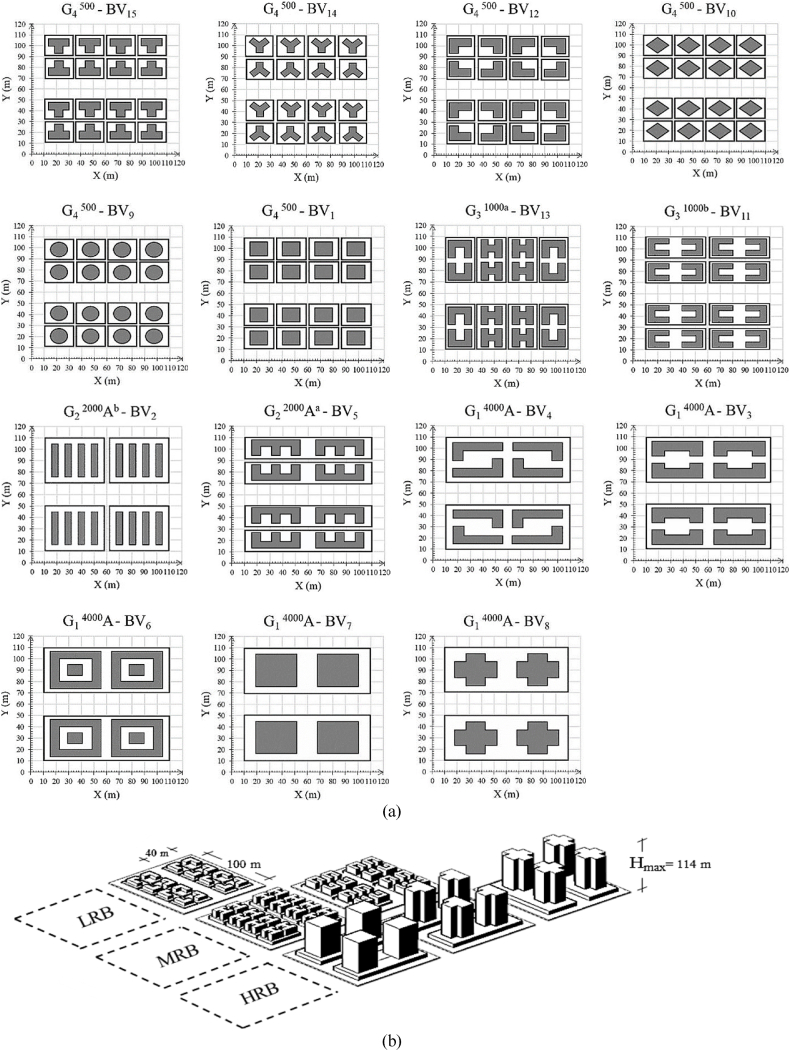
Urban design scenarios: (a) Models created within the 3D-UFO framework, showcasing different urban design configurations and (b) Diverse block configurations designed for the study.

These scenarios encompass diverse building categories, encompassing low-rise (LRB; ≤3 floors or ≤10 m in height), mid-rise (MRB; 3< floors <7, 10 m < height <24 m), and high-rise buildings (HRB; >24 m in height or residential buildings exceeding 27 m) based on building form preferences. These scenarios, or models, underwent horizontal and vertical division by 20 m roads, as depicted in Fig. 4a. Notably, following Wuhan's Building and Planning Regulations (BPR) for sites smaller than 5000 m^2^, all tall buildings designed with five-story platforms. This approach, predominantly implemented in Wuhan, accommodates diverse spaces such as malls, supermarkets, and other amenities. A typical high-rise building in our study context comprises 33 stories, corresponding to a height of 99 m (Fig. 4b). This height aligns with the average of the 386 high-rise buildings meticulously analyzed within the Wuhan urban landscape. Overall, our approach to creating 3D-UFO design strategies is methodical and rigorous. It encompasses functional zoning, plot ratios, and diverse building forms, culminating in 15 distinct scenarios towards the study objectives in densely populated Wuhan, aligning with regulatory and socio-economic factors.

## 3 Methodologies

Long-term analysis of Land-use and Land-cover Change (LULCC), in conjunction with meteorological data, conducted to investigate modifications in urban morphologies and climatic conditions. Computational Fluid Dynamics (CFD), utilizing ANSYS FLUENT 17.1, employed to simulate and assess airflow within diverse building scenarios within the framework of the 3D Urban Form Optimization (3D-UFO) approach, with the objective of optimizing urban block-scale ventilation. The analysis adhered to established urban airflow study guidelines [[49], [50], [51], [52]], ensuring scientific rigor and replicability. This comprehensive approach forms the basis for a systematic exploration of evolving urban environments, climatic dynamics, and urban design responses over time.

### 3.1 K-ω SST model and simulation domain

The k-ω SST (shear-stress transport) model, a hybrid of the k-ε and k-ω models introduced by Menter [53], was employed in this study. Specifically, the k-ω model was utilized for near-wall boundary layers, while the k-ε model was reserved for wake zones located away from the wall boundary. This model has demonstrated a remarkable capacity to accurately predict airflow patterns and wind speed distribution around the buildings, as evidenced by Zhong, Jing [54]. Consequently, it was employed for investigating vortex shedding around buildings and the eddy flow through openings.

**Fig. 5:**
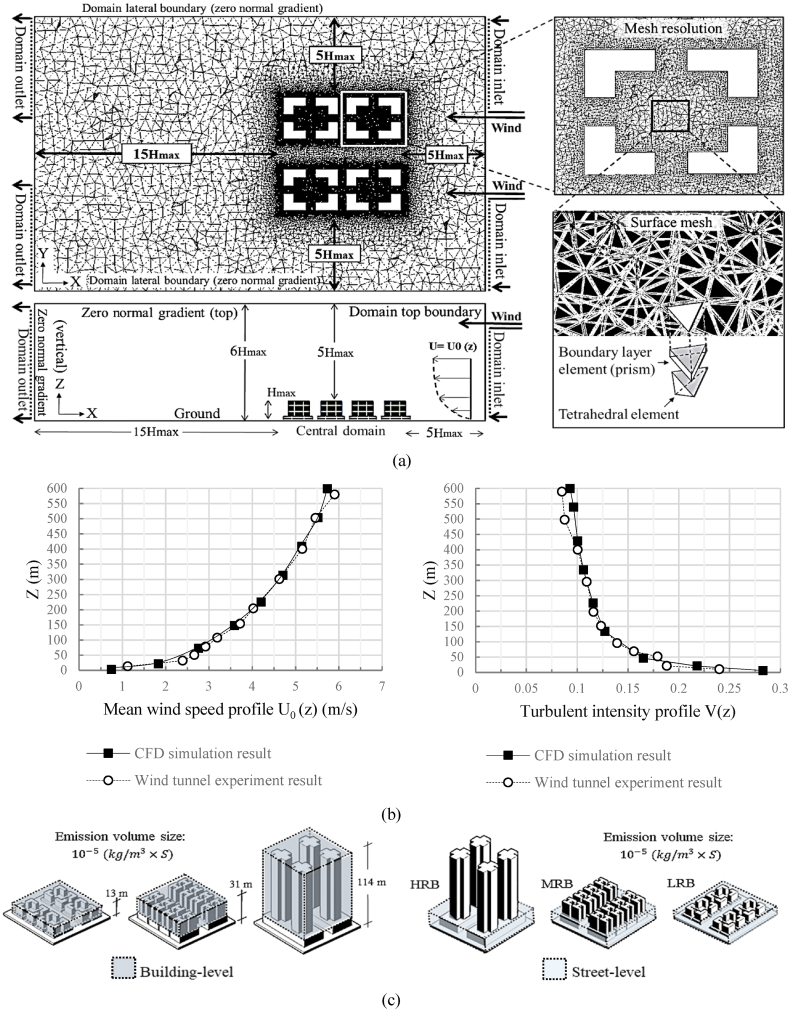
(a) Arrangement of computational domain including top and side views, mesh resolution, and surface mesh details; (b) Inlet boundary conditions showing profiles of mean wind speed and turbulent intensity; (c) Uniform source distribution at SBLs.

The computational analysis simulation domain adhered to established guidelines, depicted in Fig. 5a. It maintained a blockage ratio of less than 3 %, a dimension determined through wind tunnel experiments [49,55]. The dimensions of the top, lateral, and inlet boundaries for each domain model were adjusted to 5 H_max_ based on the target building's height (H_max_) [50,56]. In this context, H_max_ varied, with values of 13, 31, and 114 m for low-rise (LRB), mid-rise (MRB), and high-rise (HRB) buildings, respectively. Consequently, three distinct domain sizes were established: LRB = 360 × 230 × 78, MRB = 720 × 410 × 186, and HRB = 2380 × 1240 × 684 m³.

A thorough analysis involved evaluating airflow from three directions: North (N, 360°), North-northeast (NNE, 22.5°), and Northeast (NE, 45°). To account for a wide range of inbound wind conditions, 32 inbound wind directions were computed, each differing by an increment of 11.25°. This approach aligned with Wuhan's recommended air ventilation guidelines (http://hz.hjhj-e.com/home/meteorologicalData/windRose). The cases were configured to accommodate an asymmetrical design based on the prevailing wind direction, determined as NNE.

### 3.2 Establishing boundary layers

This study assessed wind availability in Wuhan using the annual mean wind speed of 2.8 m/s recorded at Jiangxia's weather station as a reference. Adhering to the guidelines set forth by the Chinese wind code [57,58], we considered the wind velocity distribution of terrains B, F, and G, as delineated by Touma [59].

Profiles of inflow streamwise wind speed (U_0_ (z) = U_0_ (z/H)^0.30^) and turbulence intensity (V(z) = 0.08227 × z^−0.206^) were established based on power laws derived from experimental wind tunnel data [54].

Conforming to the AIJ guideline [49], we characterized the specific dissipation rate ω (z), the turbulent dissipation rate ε (z), and the vertical distributions of turbulent kinetic energy k (z) as follows:

(1) $$k\;\left(z\right)=\frac{\sigma_{u\;}^{2}\left(z\right)+\sigma_{v\;}^{2}\left(z\right)+\sigma_{w\;}^{2}\left(z\right)\;}{2}≅\sigma_{u\;}^{2}\left(z\right)={\left(V\left(z\right)\;U_{0}\left(z\right)\right)}^{2}$$

(2) $$E\;\left(z\right)≅P_{k}\;\left(z\right)≅-U_{0}^{\prime }\;w^{\prime }\;\frac{dU_{0}\;\left(z\right)}{dz}≅C_{\mu }^{\frac{1}{2}}\;k\;\left(z\right)\;\frac{dU_{0}\;\left(z\right)}{dz}$$

(3) $$\omega \;\left(z\right)=\frac{E\;\left(z\right)}{k\;\left(z\right)\;C\mu }$$

Here, *σu*, *σv*, and *σw* represent the root mean square (RMS) values of velocity fluctuations in the x, y, and z directions, respectively. In the *k* equation, the production term is denoted as *Pk*, with *C*_*μ*_ (=0.09) serving as the model constant. Applying Eq. (1) and Eq. (3) allowed us to derive inlet turbulent boundary conditions for *k* *(z)* and *ω* *(z)* in accordance with the SST k-ω turbulence model.

No static pressure was imposed for the outlet boundary conditions (=0). Notably, we incorporated improved wall/surface boundary conditions, as provided by ANSYS' ICEM, to the ground, top, and building walls within the study domain [60]. Fig. 5b demonstrates the inflowing profiles of mean wind speed and turbulence intensity.

### 3.3 ALA and ASR for assessing ventilation efficiency

The Average-Age-of-Local-Air (ALA) and Airspeed Ratio (ASR) were employed as prospective metrics to assess urban ventilation efficiency. ASR, represented as α_ASR_ in Eq. (4), quantifies pedestrian-level airspeed accessibility and was calculated following Wuhan's guidelines [61].

(4) $$\alpha_{ASR}=\frac{{W\;}_{air\;speed\;at\;1.5\;m}}{{Wr\;}_{Initial\;air\;speed\;}}$$

*W*, the pedestrian-level airspeed, was determined at 1.5 m above ground in an urban environment. The initial airspeed, *Wr*, representing the reference wind velocity at the boundary layer's upper limit, customarily 350–650 m above the city's central area [54]. Here, we employed a *Wr* = 5.47 m/s at z = 500 m above the city center, as calculated by Eq. (5).

(5) $$U_{0}\left(z\right)=2.8\;{\left(\;\frac{z}{90}\;\right)}^{0.30}$$

The ALA, a pivotal parameter, reflects the concentration of airborne pollutants across various segments of a building. We rigorously calculated ALA by applying Etheridge's robust uniform emission theory [62], considering emissions from two distinct sources: Nitrogen Dioxide (NO_2_), predominant vehicular-derived pollutant at street-level, and H_2_O molecular diffusion at building-level, spanning the region from street-level to beneath the urban canopy. To estimate ALA precisely, we employed the following equation:

(6) $${\bar{⍺}}_{ALA}=\frac{\bar{P}\;\left(time\right)}{P_{e}\;\left(kg/m^{3}\times S\right)}$$

Where ${\bar{\alpha }}_{ALA}$ represents the air turnover time, reflecting its age, $\bar{P}$ signifies the pollutant concentration over time, and $P_{e}$ denotes the pollutant emission volume size, set at $10^{-5}\;\left(kg/m^{3}\times S\right)$ [63]. Utilizing Eq. (6), we calculate air turnover time within the urban canyon, including both street and building levels (SBL_S_). Fig. 5c illustrates the volume space of the SBLs.

### 3.4 Computational grid resolution and independence testing

In this study, we utilized a standardized mesh with optimized resolution to fine-tune our models, as presented in Fig. 6a, Fig. 6ba, providing an isometric view of the models with their surface meshes. The smallest surface mesh dimension, at 0.3 m, accurately captured the geometric intricacies of the building's ground, corners, and top grid. Grids located farther from the models used larger mesh sizes. Employing an extension ratio of 1.1, as proposed by Tominaga, Mochida [49], allowed us to effectively adapt grid sizes for adjacent cellular units.

**Fig. 6a:**
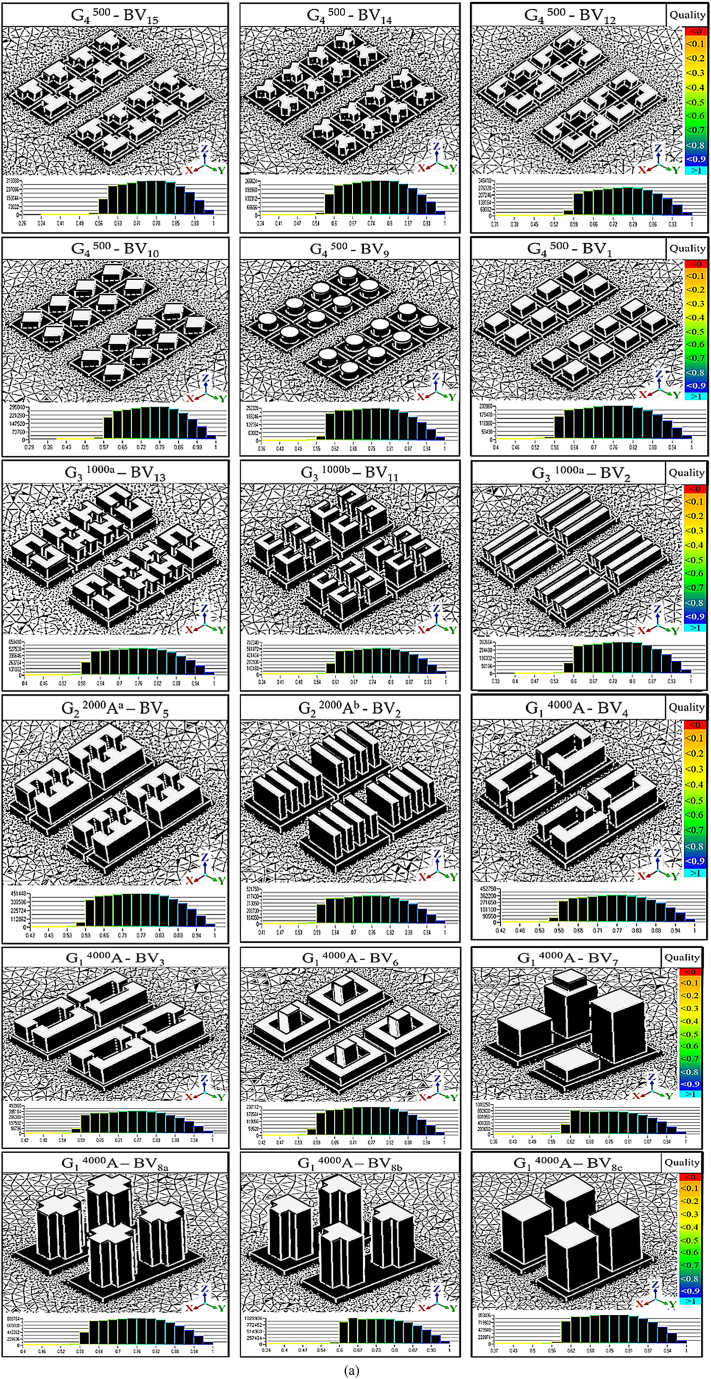
Mesh Quality and Quantity Analysis **(a)** Isometric depiction of domain meshes across models, color-coded to indicate quality levels from low (<0) to high (>1). Green highlights regions exceeding the 0.3 quality threshold, indicating superior segments.

With the 3D-UFO approach, we established eighteen distinct models to examine various urban building forms and site scenarios thoroughly. Fig. 6b details the total tetrahedral grid elements for all models, with variations in cell size corresponding to the diverse geometrical features of each model. Grid independence was assessed by conducting grid convergence tests following the methodology outlined by Celik et al. [64].

**Fig. 6b:**
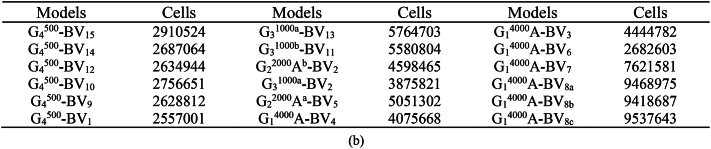
Total number of tetrahedral grid elements for all models represented as cells.

### 3.5 Optimal turbulence model selection

In pursuit of optimal turbulence modeling, we employed RANS turbulence models with dual precision to address steady-state turbulent flow challenges in ANSYS FLUENT 17.1, maintaining an isothermal environment. The assessment included various RANS-based turbulence models, including Reynolds stress (RS) [65], Standard k-ω (k-omega) [66], Standard k-ε (k-epsilon) [53], Realizable k-ε (RK-epsilon) [67], RNG k-ε (RNG k-ε) [68], and Shear stress transport k-ω (SST k-omega) [69]. The analysis aimed to identify the most suitable model and determined the SST k-omega turbulence model as the optimal choice due to its superior reliability and precision. Section 4.3 delves deeper into the nuances of model validation.

## 4 Model validation

### 4.1 Empirical data analysis

**Fig. 7:**
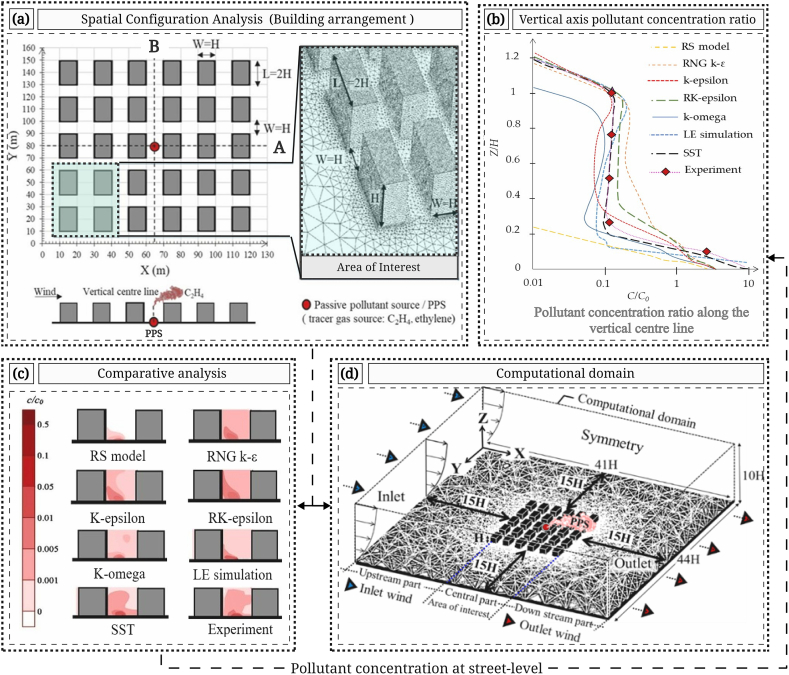
Analysis of Urban Air Quality and Computational Domain: (a) Measurement of building arrangement (left) and area of interest on building blocks (right); (b) Ratio of pollutant concentration along the vertical axis; (c) Comparative analysis of pollutant concentration at street-level; (d) Dimensions of the computational domain.

Empirical wind tunnel tests conducted by Tominaga and Stathopoulos [70] and An and Fung [69], emphasizing close-surface pollutant dispersal, served for validating CFD simulations and examining block configuration outcomes. These tests were performed in a boundary layer wind tunnel measuring 1.8 × 1.8 × 13 m (width, height, and length). Boundary conditions and surface roughness were harmonized with a power-law exponent to simulate an approaching wind profile. Within this controlled environment, a passive pollutant source (tracer gas: C_2_H_4_) was emitted at the street-level's base (Fig. 7a) with a concentration of one thousand parts per million. Wind speed was measured using a split-film probe, and pollution/gas concentrations were quantified via a fast-response flame ionization detector at a 1000 Hz sampling frequency, yielding 120,000 data records in 120 s. To replicate an urban context, cuboid building blocks were arranged near the pollutant source. Fig. 7b depicts specific variations in pollutant concentration ratios along the vertical centerline. For further test data insights, review Tominaga and Stathopoulos [70], Tominaga and Stathopoulos [71], and An and Fung [69].

### 4.2 Setup of computational domains and boundaries

The computational domain, as illustrated in Fig. 7d, faithfully replicates the building block configurations as defined in the wind tunnel tests. Adhering to CFD guidelines established by Tominaga, Mochida [49], which stipulate the necessity of situating the lateral and upper boundaries at distances ≥15H from the target building (with H denoting the height of the tallest structure), shaping the domain's dimensions in H-scale along the X, Y, and Z axes. Blockage ratios for the domain models were consistently below 3.0 %, averaging approximately 2.2 %. Boundary conditions for the CFD simulations were derived from the experimental wind tunnel study, while the choice of the k-ω SST turbulence model aligned with analytical and modeling preferences.

The SST model's specific form is computed as follows:

(7) $$\frac{\partial \left(\rho k\right)}{\partial t}+\frac{\partial \left(\rho u_{j}k\right)}{\partial x_{j}}=P_{1}-\beta^{*}\rho \omega k+\frac{\partial }{\partial x_{j}}\left[\left(\mu +\sigma_{k}\mu_{t}\right)\frac{\partial k}{\partial x_{j}}\right]$$

(8) $$\frac{\partial \left(\rho \omega \right)}{\partial t}+\frac{\partial \left(\rho u_{j}\omega \right)}{\partial x_{j}}=\frac{\gamma }{v_{t}}P_{1}-\beta \rho \omega^{2}+\frac{\partial }{\partial x_{j}}\left[\left(\mu +\sigma_{k}\mu_{t}\right)\frac{\partial \omega }{\partial x_{j}}\right]+2\left(1-F_{1}\right)\frac{\rho \sigma_{\omega 2}}{\omega }\;\frac{\partial k}{\partial x_{j}}\;\frac{\partial \omega }{\partial xj}$$

The variable ω is defined as:

(9) $$\omega =\frac{\beta^{*0.25}k^{0.5}}{\beta^{2}}$$

Where *β2* = 0.07 and β* = 0.09 represent the model constants for the SST k-ω model.

The experimental setup involved ethylene (C_2_H_4_) as a tracer gas, covering 0.01H^2^ at a height of 0.25H with a volumetric flow rate of 5.83 × 10^−6^ m^3^/s, as specified by Tominaga and Stathopoulos [70] and An and Fung [69]. This research used wind tunnel-derived data, adopting information from an experiment of flow and pollutant concentration around buildings.

### 4.3 Comparative analysis and model affirmation

A comparative assessment of RANS turbulence models, namely RS, k-omega, k-epsilon, RK-epsilon, RNG k-ε, and SST k-omega, was conducted (Fig. 7c). The analysis relied on data from the cited wind tunnel experiments and large-eddy (LE) simulations [70]. The results of the RANS turbulence models exhibited strong concordance with wind tunnel and large-eddy simulation findings, particularly in relation to wind speed ratios and pollutant concentrations with respect to U_0_ (z). Among these models, SST k-omega displayed an average standard deviation (STDEVA) that closely approximated RK-epsilon and RNG k-ε, around 0.22. In contrast, it was lower compared to SKE, SKW, and RSM concerning the wind speed ratio (SST k-omega, RK-epsilon, RNG k-ε ≅ 0.22 < SKE, SKW, RSM). During the evaluation of the street centerline concentration of pollutants on the vertical axis, SST k-omega displayed a significantly lower standard deviation (STDEVA = 0.009) when compared to all other RANS turbulence models. Due to its accuracy and strong alignment with experimental results, the SST k-omega model was chosen for the present study.

### 4.4 Mesh refinement in computational models

As described in section 3.4, a 0.3 m mesh size was imposed to capture geometric intricacies. To ascertain that alterations in mesh intricacy do not influence simulation results, models were subjected to mesh refinement. Various grid types—high-resolution (19,075,286 grids, min mesh 0.1 m), medium-resolution (6,358,428 grids, min mesh 0.3 m, ideal), and low-resolution (2,725,040 grids, min mesh 0.7 m)—were evaluated for sensitivity. Uniform solver setups and computational domains were utilized for comparative analysis of airflow and pollutant dispersion at 1.5 m above ground, along planes A and B (Fig. 10a, Fig. 10b). Error analysis revealed minor distinctions between medium-resolution and high-resolution meshes, but significant divergences emerge between high-resolution and low-resolution meshes. Notably, in the A-plane (Fig. 10a, Fig. 10b), medium-resolution records NO_2_ concentration at 0.17, while high-resolution records NO_2_ = 5.12. Consequently, the validation of a medium-resolution grid (min mesh = 0.3 m) is confirmed for this study.

## 5 Results and discussion

### 5.1 Street-level airflow efficiency

Urban street-level airflow efficiency relies on two key metrics, ALA and ASR. Fig. 8a, Fig. 8b illustrates air age distributions at 1.5 m above the terrain, accompanied by the corresponding ASR values for each scenario. Fig. 9 in Subfigure A delineates average ALA characteristics observed at street-level (Fig. 9a) and within building-level (SBLs) contexts (Fig. 9c), complemented by an analysis of volume-averaged fundamental ventilation modes. Additionally, it examines the average ASR at specified points (designated in Fig. 10c), contributing to a comprehensive understanding of local atmospheric dynamics. Fig. 9 in Subfigure B further illustrates key parameters, including average momentum, dynamic pressure on buildings and the ground, average ALA at building facades, and momentum averages for all cases. Spatial ASR distributions closely mirror airflow patterns influenced by specific building arrangements. Optimal urban air circulation, achieving efficiency levels of 50–70 %, is attained when airflow aligns orthogonally across and parallel to street lanes. Our findings emphasize that increasing street width within larger sites enhances air mixing, resulting in augmented air circulation. Moreover, varying street-level building heights, particularly with taller structures, significantly enhances ventilation by 20–30 %. The street aspect ratio (H/W, building height/street width) exerts a profound influence on airflow enhancement and pollutant dispersion within urban streets, in close interaction with surface features like roofs and facades. Higher H/W ratios, for instance, H/W > 5.5, produce a remarkable 218.5 % enhancement in air access and ventilation. Street geometry, H/W ratio, and street orientation significantly influence wind control, thereby affecting wind energy harvesting from street surfaces. Therefore, the arrangement of urban patterns is pivotal.

**Fig. 8a:**
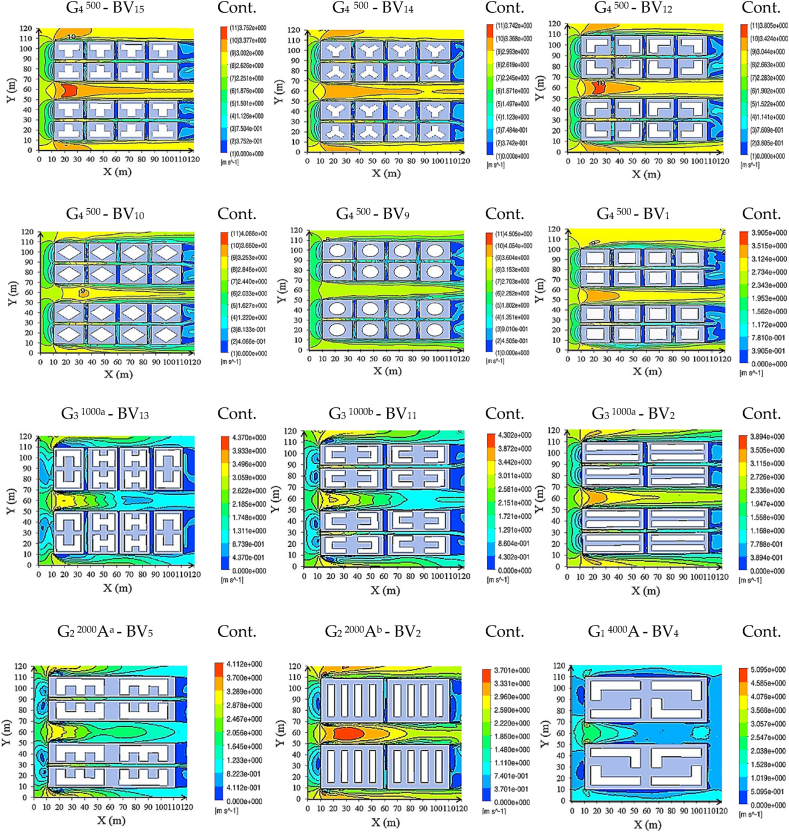
Air age distributions at street-level (1.5 m above terrain) influenced by incoming dominant wind.

**Fig. 8b:**
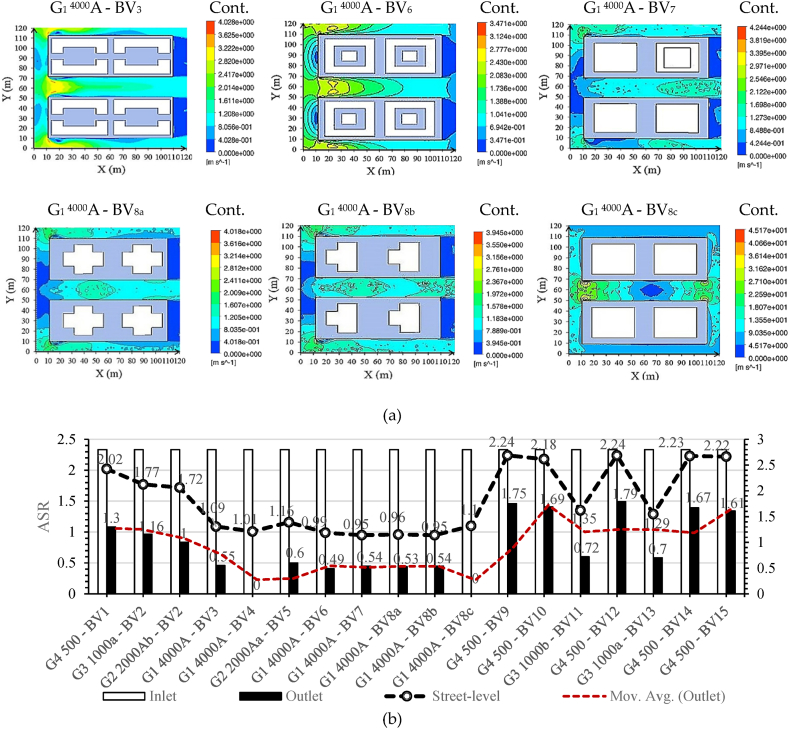
Average volume of wind velocity/ASR at street-level (m/s).

**Fig. 9:**
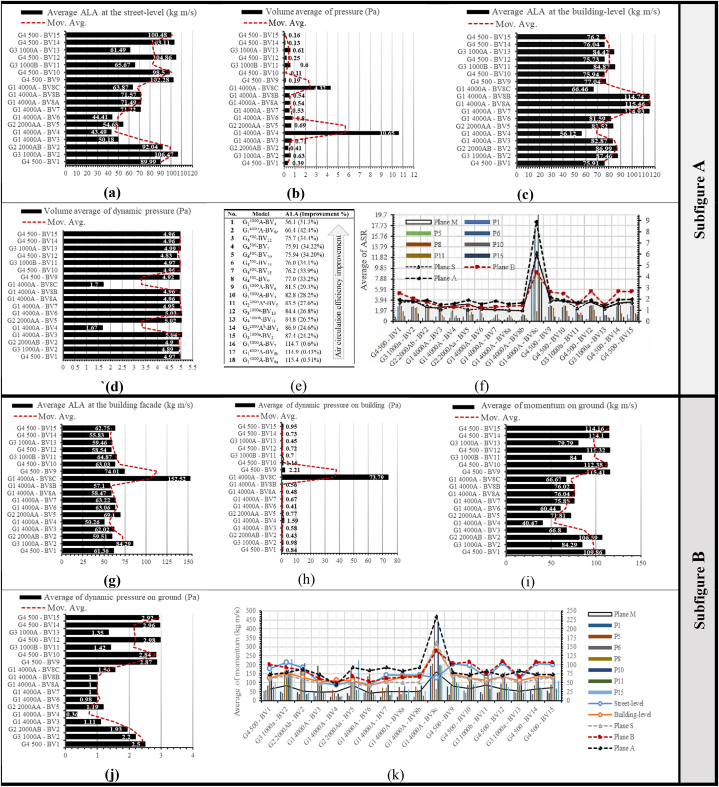
Performance Metrics and Airflow Characteristics: Subfigure A encompasses (a) Average ALA at the street-level (kg m/s), (b) Volume average of pressure (Pa), (c) Average ALA at the building-level (kg m/s), (d) Volume average of dynamic pressure (Pa), (e) Performance improvement of models, and (f) Average ASR in specified points and planes at the building-level (m/s). Subfigure B comprises (g) Average ALA at the building facade (kg m/s), (h) Average of dynamic pressure on building (Pa), (i) Average of momentum on the ground (kg m/s), (j) Average of dynamic pressure on ground (Pa), and (k) Average of momentum in specified points and planes at the building-level (m/s).

On average, all models display an ASR of 1.52 during prevailing wind conditions. Specifically, when the prevailing wind aligns with the main street grid, we observe substantial wind velocity reductions in G_1_^4000^A-BV_6_ (53.5 %), G_1_^4000^A-BV_4_ (55.3 %), and G_1_^4000^A-BV_7_ (56.0 %) cases. At street level, the average ALA volume was assessed across sites featuring low-rise, mid-rise, and high-rise buildings, yielding an average of 77.5. Notably, mid-rise sites recorded the lowest average at 64.8 under prevailing wind conditions. However, this value increased to 69.5, marking a 12 % rise in high-rise sites, and significantly higher at 99.8, indicating a substantial 51 % increase in low-rise sites when the incoming wind direction remained constant.

The study divulges intriguing aerodynamic insights. Specifically, it unveils a marginal ASR variance between inlet and outlet winds, denoting a value of 1.01 (G_4_^500^-BV_12_). In stark contrast, G_1_^4000^A-BV_8c_ and G_1_^4000^A-BV_4_ exhibit the most marked difference, presenting a ratio of 2.8. An astounding wind velocity increase of 218.5 %, more than double the baseline, observed when the H/W ratio exceeds 5.5 and the prevailing wind aligns orthogonally with the street grid at 90°. This significant enhancement in wind velocity is primarily attributed to the incorporation of high-rise buildings with H/W > 5.5, especially those featuring simple cubic shapes and medium-density ratios, which markedly improve airflow within the urban canyon.

In this context, high-rise buildings with uncomplicated cubic configurations and medium-density proportions exhibit the highest wind velocity, intensifying the wind speed on the central street by 1.5 times. Consequently, the inclusion of high-rise structures augments canyon airflow, mitigates temperatures, and aids air pollutant dispersion within urban environments. Investigation reveals that fine-tuning the H/W ratio (shallow = H/W < 0.5, deep = H/W ≥ 2, and regular H/W = 1) unlocks a 35 % increase in avenue canyon wind velocity. At the street-level, to ameliorate concentrations of pollutants, particularly elevated in the region between the pollutant source and the leeward building facade, an H/W ratio adjustment to 1/2 is recommended.

Summarily, in urban environments like Wuhan and various other cities grappling with the transformation of street grids, the evolution of site designs and building configurations emerges as the central element shaping air dynamics in pedestrian zones. Buildings with H ≤ W, marked by the gradual widening of their structures, lead to decreased wind velocity near the building facades. This diminishment is especially pronounced in the central segments of the vertical planes adjoining the building facades (see fig. 10c for detailed plane analysis). Notably, wind speed values at the top and lateral edges remain virtually identical across all cases. In the case of buildings with H ≥ W, characterized by heightened structures, wind velocities at the edges experience acceleration.

In proximity to the building bases, an area exhibits diminished wind speeds, wherein the horseshoe vortex governs the airflow. A closer examination of the simulated patterns reveals that the height of the horseshoe vortex remains relatively constant regardless of the building's height. Maximum ASR values transpire at the top and lateral edges, where wind speeds are notably elevated. Subsequently, the elevation of building heights amplifies the maximum ASR values. In effect, a uniform ASR distribution across the central region of the facades becomes evident, representing uniform zones of low velocity and high pressure. The area characterized by the horseshoe vortex's formation displays lower ASR values, attributed to the rotational nature of the vortex, which prolongs air residence time in this locale, consequently raising the local air temperature and reducing local ASR values. Buildings with H = W also exhibit higher local ASR as their sizes increase, especially near the lateral and top edges. Examining S-plane data in Fig. 10c uncovers substantial average wind velocity reductions, particularly at 35 % (G_1_^4000^A-BV_8b_), 35.2 % (G_1_^4000^A-BV_8a_), 36.7 % (G_1_^4000^A-BV_3_), and 38.5 % (G_1_^4000^A-BV_7_).

**Fig. 10a:**
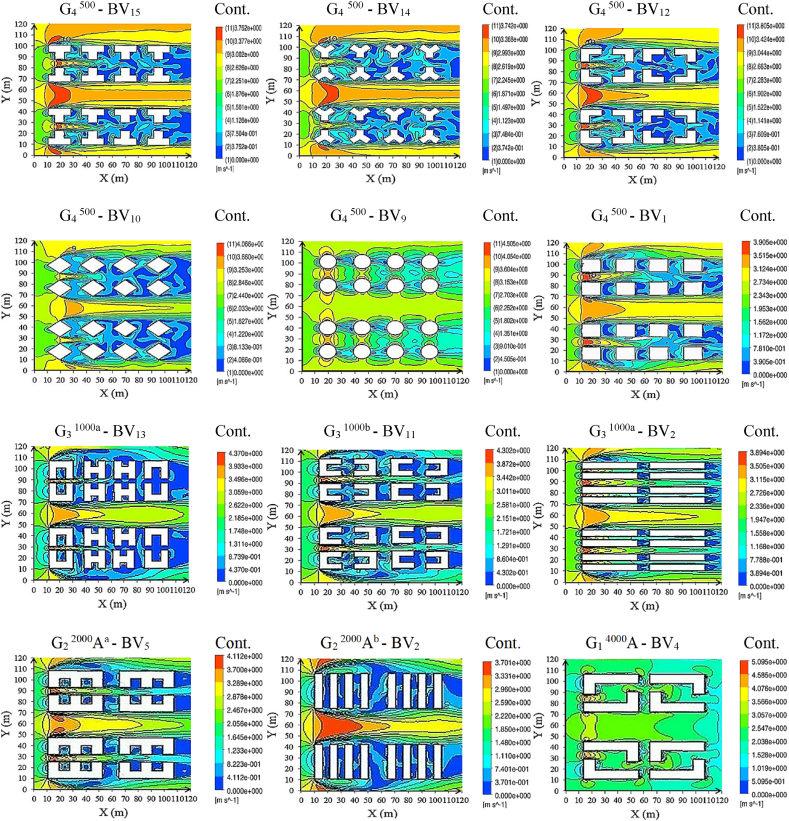
Building-Level Airflow Analysis: (a) Air age distribution at the building-level influenced by dominant incoming winds;

**Fig. 10b:**
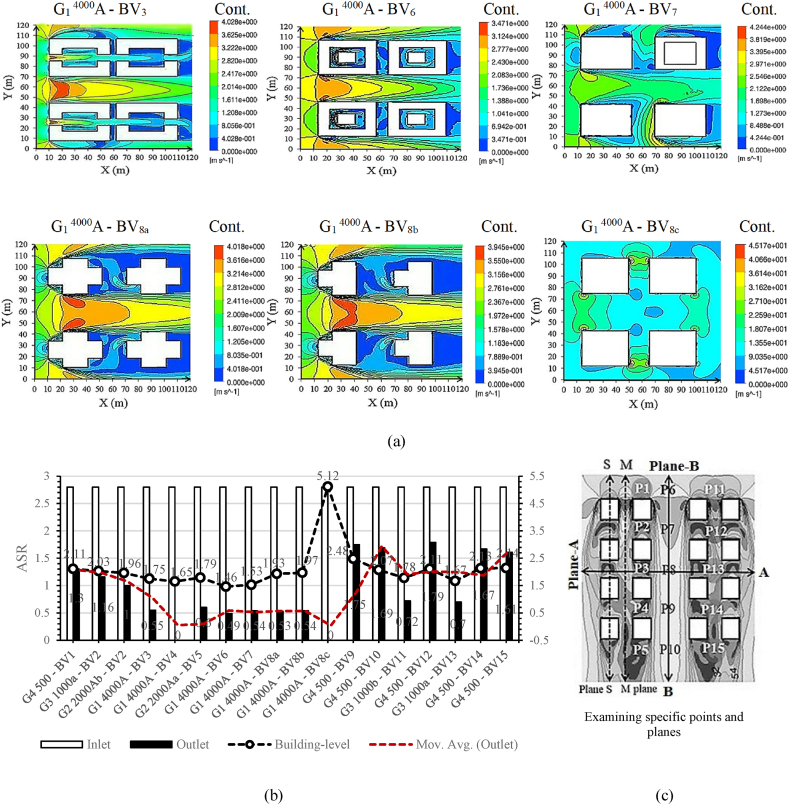
Volumetric analysis of ASR volumetric analysis at the building-level; (c) Detailed analysis at specific planes and points.

These findings illuminate the pivotal role of pressure differentials as the primary governing mechanism dictating airflow dynamics across building surfaces. This phenomenon comes to the forefront, particularly in the domain of wind pressure-driven ventilation, highlighting variances contingent upon the intricate interplay of building morphology. Optimizing synergistic relationships within site configurations, particularly the interactions between building structures and their surroundings through 3D-UFO, promotes efficient natural resource utilization and augments local wind environments. This meticulous site design enhances wind energy harvesting and reinforces sustainable urban ventilation strategies. Subsequent observations emphasize the vital importance of maintaining a minimum wind velocity threshold of 0.3 m/s to optimize aerodynamic turbulence, which promotes efficient air mixing. This optimization significantly enhances convective airflow dynamics, thereby contributing to the dispersion of airborne particulates. Our findings affirm that medium-to-high height-to-width (H/W) ratios robustly enhance air velocities and foster turbulent eddies within the urban canyon, thereby improving atmospheric quality and local thermal conditions at pedestrian levels. Heightened building density in narrow street configurations sharply curtails airflow, leading to marked reductions in flow velocity, momentum, turbulent kinetic energy, and limiting flow adjustments. This coincides with reduced in-canopy airflow in the fully developed region. In contrast, wider streets and lower building density, fueled by enhanced spatial airflow exchange, significantly bolster vertical ventilation capacity. The G_4_^500^-BV_9_ site, characterized by cylindrical building obstacles oriented at a 90° angle to the incoming wind, boasts the highest inflow wind velocity, coupled with the most minimal wind velocity reduction at 37.1 %. The heightened coefficient of variation underscores that irregular or rough surfaces engender amplified turbulent flow and increased flow rate variability, attributed to intensified chaotic motion of fluid molecules. This effect, despite maintaining identical mean flow rates, results in a more conspicuous reduction in wind speed at a height of 1 H and a less pronounced decrease at a height of 1.5 H from the obstruction, extending over a horizontal distance of 5 H, as compared to the impact of smooth surface turbulent flow.

Findings indicate that projects situated on expansive sites offer the most effective street-level ventilation when paired with taller structures. Among the large sites under examination, G_1_^4000^A–BV_4_ and G_1_^4000^A–BV_6_ exhibit superlative street-level ventilation performance across a spectrum of prevailing wind directions, demonstrating an impressive minimal average ALA at 43.4 (ASR = 1.01) and 44.4 (ASR = 0.99). Conversely, G_1_^4000^A-BV_8b_, G_1_^4000^A-BV_8a_, G_1_^4000^A-BV_7_, and G_1_^4000^A-BV_8c_ exhibit inferior street-level ventilation efficiency, despite featuring tall buildings and identical site division scheme. Furthermore, G_3_^1000a^-BV_2_, characterized by a smaller site and mid--rise linear buildings, presents the highest ALA yet the poorest street-level ventilation.

Utilizing the average impact index ($\rho_{v}=\frac{\sum \beta_{v}\mu_{v}}{N_{v}}$), where *β*_*V*_ signifies the variable mean, *μ*_*V*_ represents each variable's coefficient, and *N*_*V*_ denotes the count of observations for each variable, G_4_^500^–BV_1_ emerges as the optimal model. This model excels in promoting street-level ventilation in smaller sites dominated by low-rise buildings. In the context of larger sites featuring taller structures, models G_1_^4000^A-BV_4_ and G_1_^4000^A-BV_8c_ effectively contribute to the improvement of urban ventilation. Nevertheless, for midsize and extensive site layouts typified by G_1_^4000^A–BV_3_, G_2_^2000^A^a^–BV_5_, G_3_^1000a^–BV_13_, and G_3_^1000b^–BV_11_, which are characterized by fragmented mid-rise H and U building forms, the observed ventilation levels remain relatively moderate. This observation underscores that densely packed urban plans within small and medium-sized projects experience constrained street-level ventilation in comparison to more spacious layouts featuring larger open areas.

In contrast to regular high-rise building forms like G_1_^4000^A-BV_8b_ and G_1_^4000^A-BV_8a_, irregularly shaped high-rise structures (G_1_^4000^A-BV_7_) integrated with mid-rise and low-rise buildings with extensive layouts improve street-level ventilation by approximately 0.5 %. Accordingly, projects with typical mid-rise buildings could potentially amplify this effect by 8.24 % (G_3_^1000b^-BV_11_), 14 % (G_3_^1000a^-BV_13_), 23.5 % (G_2_^2000^A^a^-BV_5_), 29.8 % (G_1_^4000^A-BV_3_), 37.9 % (G_1_^4000^A-BV_6_), and 39.2 % (G_1_^4000^A–BV_4_) at street-level.

In the majority of cases, buildings characterized by higher floor area ratios exhibit negligible positive impact on the enhancement of street-level ventilation, particularly in smaller sites. Concurrently, the adoption of small site-divisional strategies featuring service lanes demonstrates a pronounced efficacy in augmenting street-level ventilation. A comparative analysis between G_2_^2000^A^a^-BV_5_ and G_1_^4000^A-BV_8a_ validates this outcome. Examination of diverse high-rise block configurations, including G_1_^4000^A-BV_8a_, G_1_^4000^A-BV_8b_, and G_1_^4000^A-BV_8c_, affirms that distinct building forms represent the paramount factor influencing air dynamics at street-level. However, disparate building forms within the spectrum of low-rise, mid-rise, and high-rise structures manifest varying effects on ventilation enhancement through the strategic utilization of service paths.

### 5.2 Building-level airflow efficiency

Within the 3D-UFO framework, our investigation delved into building-level airflow efficiency, shedding light on airflow effectiveness from above street-level to a height of 114 m. Fig. 10 visually represented air age distributions within the average building heights above pedestrian levels, concurrently with observed ASR under prevailing wind conditions. Acknowledging ALA's pivotal role in assessing air quality near buildings for residents' welfare, we calculated its building surface mean by integrating air age values across each facet. All models exhibited an average ALA of 84.2 under prevailing wind conditions. Further analysis revealed a compatible relationship between building-level and street-level air circulation when the angle of the prevailing wind and the central street grid increased. Fig. 9 in Subfigure A featured an exhaustive analysis of critical ventilation modes, coupled with ALA and ASR at the building-level. Notably, G_1_^4000^A-BV_8a_ exhibited the highest average ALA at 115.4 (100 %).

Subsequently, G_1_^4000^A-BV_8b_ achieved 114.9 (0.43 % improvement), G_1_^4000^A-BV_7_ reached 114.7 (0.6 % improvement), G_3_^1000a^-BV_2_ attained 87.4 (24.2 % improvement), G_2_^2000^A^b^-BV_2_ revealed 86.9 (24.6 % improvement), G_3_^1000b^-BV_11_ displayed 84.8 (26.5 % improvement), G_3_^1000a^-BV_13_ exhibited 84.4 (26.8 % improvement), G_2_^2000^A^a^-BV_5_ exposed 83.5 (27.6 % improvement), and G_1_^4000^A-BV_3_ demonstrated 82.8 (28.2 % improvement). G_1_^4000^A-BV_6_ achieved 81.5 (29.3 % improvement), G_4_^500^-BV_9_ recorded 77 (33.2 % improvement), G_4_^500^-BV_15_ reached 76.2 (33.9 % improvement), G_4_^500^-BV_14_ demonstrated 76 (34.1 % improvement), G_4_^500^-BV_10_ attained 75.94 (34.2 % improvement), G_4_^500^-BV_1_ revealed 75.91 (34.22 % improvement), and G_4_^500^-BV_12_ exhibited 75.7 (34.4 % improvement). Results from G_1_^4000^A-BV_8c_ (ALA = 66.4, 42.4 % improvement) and G_1_^4000^A-BV_4_ (ALA = 56.1, 51.3 % improvement) underscore enhanced urban air circulation within expansive sites (G_1_^4000^ = 4000 m^2^). Fig. 9e elucidated these outcomes.

During our meticulous investigation, elevating the H/W (height-to-width) ratio significantly diminished canyon retention time, enhancing airflow dynamics. This revelation offers an intriguing perspective on air circulation within canyons. Additionally, implementing a centralized tall building plan can enhance local air ventilation. Compared to big sites, mid-sized sites (G_2_^2000^ = 2000 m^2^) exhibited solely modest building-level air circulation. In smaller sites (G_3_^1000^ = 1000 m^2^) and more compact ones (G_4_^500^ = 500 m^2^), the probability of attaining adequate ventilation decreased as on-site building density rose. Remarkably, G_4_^500^-BV_12_ employed a segmented operational strategy for diminutive sites, yet it achieved the third-lowest average ALA volume at 75.7. This reveals that enhancing ventilation in smaller sites is achievable through the implementation of well-structured symmetrical L-shaped buildings featuring central spaces strategically designed to incorporate openings aligned with predominant wind directions.

Building-level natural ventilation extends beyond its influence on urban outdoor ventilation efficiency, concurrently exerting an impact on indoor air quality. Fig. 9g in Subfigure B provided a quantitative assessment of the indoor natural ventilation potential of each building, characterized by ALA measurements at the facade. These measurements indicate the accessibility of fresh outdoor air for indoor natural ventilation, enhancing overall air quality. Among the building configurations assessed, G_1_^4000^A-BV_8c_ achieved the highest average ALA at the building facade, an impressive 152.5 (100 %), attributed to its distinctive cuboid morphology and voluminous depth.

Subsequently, G_3_^1000a^-BV_2_ at 84.2, G_4_^500^-BV_9_ at 74, G_2_^2000^A^a^-BV_5_ at 69.1, G_3_^1000b^-BV_11_ at 64.8, G_1_^4000^A-BV_7_ at 63.2, G_1_^4000^A-BV_6_ at 63.06, G_4_^500^-BV_10_ at 63.03, G_4_^500^-BV_15_ at 62.75, G_1_^4000^A-BV_3_ at 62.02, G_4_^500^-BV_1_ at 61.3, G_2_^2000^A^b^-BV_2_ at 59.5, G_3_^1000a^-BV_13_ at 59.4, G_4_^500^-BV_12_ at 58.54, G_1_^4000^A-BV_8a_ at 58.47, G_1_^4000^A-BV_8b_ at 57.1, and G_4_^500^-BV_14_ at 55.8 demonstrated substantial air circulation improvements of 44.7 %, 51.4 %, 54.6 %, 57.5 %, 58.5 %, 58.64 %, 58.66 %, 58.8 %, 59.3 %, 59.8 %, 60.9 %, 61.04 %, 61.63 %, 61.65 %, 62.5 %, and 63.4 %, respectively.

Overall, G_1_^4000^A-BV_4_ exhibited the highest potential in ameliorating indoor air quality, characterized by a minimal 50.2 ALA at the building facade within an expansive 4000 m^2^ site, and optimal ventilation efficiency, resulting in a substantial 67 % enhancement. The investigation, grounded in 3D-UFO analysis, unequivocally revealed a robust correlation between natural ventilation capacities within interior spaces and the intricate interplay of building forms and configurations, notably accentuated in elevated constructions and high-rise block schemes within larger projects. Emphasizing the indispensable role of integrating urban building geometry and directional wind patterns under the H/W ratio for optimal design efficiency, our elucidation markedly advanced the understanding of ventilation potential across diverse site classifications. The study conclusively demonstrated the superiority of meticulous design in small-scale sites with Y-formed structures (G_4_^500^-BV_14_) for enhancing indoor natural ventilation. Subsequent observations identified this superiority in larger sites with T-shaped configurations (G_1_^4000^A-BV_8b_) and regular high-rise building plans (G_1_^4000^A-BV_8a_).

Following, G_4_^500^-BV_12_ highlighted systematically configured L-shaped construction plans in small-scale projects, revealing a 61.63 % enhancement in ventilation efficiency. This underscores the attainability of optimal airflow within enclosed spaces through a symmetrical design, integrating central courtyards and strategically positioned openings along both vertical and horizontal axes. In G_3_^1000a^-BV_13_, a symmetric U-shaped and H-shaped integration, and in G_2_^2000^A^b^-BV_2_, elongated, depth-oriented geometric configurations led to significant improvements of 61.04 % and 60.9 % in indoor natural ventilation. Across a spectrum of site scales, encompassing large to small with different building plans, G_1_^4000^A-BV_8c_ (high-rise), G_3_^1000a^-BV_2_ (mid-rise), and G_4_^500^-BV_9_ (low-rise) exhibited reduced potential for internal natural ventilation. Observations elucidated that a heightened H/W value exerted a detrimental influence on indoor natural ventilation within these scenarios, wherein turbulent airflow patterns induced, impeding the efficient interchange of indoor and outdoor air and consequently diminishing the overall efficacy of ventilation.

### 5.3 Discussion

This study employed the innovative 3D-UFO methodology to scrutinize air circulation and pollutant dispersion within densely built-up urban environments. The results realistically elucidate multifaceted outcomes and portray the challenges associated with urban pollution, offering quantitative insights into potential strategies for improving air quality and fostering healthier living conditions. Our approach, grounded in a comprehensive analysis of LULCC, coupled with CFD and systematic exploration, guided by established principles of urban airflow study protocols, facilitated robust simulation and assessment of airflow in diverse site scenarios and block configurations.

In large sites of approximately 4000 m^2^, distinguished by expansive building plans, a discernible inclination manifested toward optimizing ventilation at the building-level. Nevertheless, these sites often demonstrated limited capacity to improve indoor air circulation and generally did not assure high air circulation performance at the street-level. This observation aligned with prior findings by Zhang et al. [35]. Among large plan projects featuring high-rise buildings, G_1_^4000^A-BV_8c_ exhibited a grade A street-level ventilation performance of 10.7 %, particularly effective in a deep avenue canyon (H/W ≥ 2), ranking first in building-level ventilation (ALA = 66.4). However, it does not prioritize indoor ventilation. Contrarily, G_1_^4000^A-BV_4_ demonstrated superior building-level ventilation, ranking highest in air circulation and achieving elevated indoor ventilation.

G_1_^4000^A-BV_6_ exhibited optimal building-level air circulation performance, securing the highest rank in this aspect, and attained the second position in street-level ventilation. Notably, a limitation manifested in its indoor ventilation, characterized by suboptimal conditions. This observation aligned with insights from Antoszewski's study [34], wherein various contributing factors, including the strategic positioning of elevated central buildings with a relatively modest frontal area, played a pivotal role in shaping air circulation dynamics within urban settings. Therefore, implementing a strategy for centrally located elevated buildings with a relatively small frontal area and preventing deep spaces with enclosed plans, particularly under 3D-UFO, can promote airflow within the urban context. These findings resonate with those by Nugroho et al. [72], which explored how geometric parameters (such as form and dimensions) and volumetric characteristics influence the thermal microclimate around high-rise structures, thereby enhancing our understanding of how spatial configurations impact airflow patterns and environmental quality in dense urban settings.

Our 3D-UFO study revealed that additional service paths enhanced street-level ventilation in small and medium-sized sites (approximately 500 m^2^, 1000 m^2^, and 2000 m^2^). Conversely, such strategies may lead to limited ventilation at building-level in larger site projects. For instance, G_2_^2000^A^a^-BV_5_ ranks fourth in street-level and mid-rank building-level ventilation in mid-sized projects. Small and medium-sized sites may employ distinct strategies, such as small floor plans and elevated structures along block axes, to optimize building-level air circulation and indoor ventilation, unlike service path approaches. The most reliable examples for sites with low-rise buildings were G_4_^500^-BV_14_ and G_4_^500^-BV_12_, each characterized by superior indoor air circulation and satisfactory building-level ventilation, albeit with comparatively less dependable street-level ventilation. Within this classification, G_4_^500^-BV_1_ manifested the most delicate air circulation at street-level, whereas G_4_^500^-BV_10_, concurrently attaining the second-highest rating in street-level air circulation, demonstrated the most optimal ventilation at the building-level.

In sites with mid-rise buildings, G_3_^1000a^-BV_2_ ranked first in poor ventilation at street-level, building-level, and indoor air quality compared to mid-sized sites (1000–2000 m^2^). Following a discernible airflow pattern, G_3_^1000a^-BV_2_ ranked in the lower tertile, demonstrating analogous poor ventilation at street-level, building-level, and indoor air quality.

Ventilation degradation, eliciting adverse effects, exhibited amelioration at both street and building levels (SBLs) within larger sites (G_2_^2000^). Notably, mid-rise structures, exemplified by G_2_^2000^A^a^-BV_5_, secured third and fourth rankings in this enhancement paradigm. G_2_^2000^A^b^-BV_2_ exhibited penultimate suboptimal ventilation at SBLs, attributed to its profound avenue canyon (H/W = 4 ≥ 2). Jamei and Rajagopalan [73] expounded this intricacy, emphasizing the connection between air temperature within deep canyons and the magnitude of the H/W ratio. This denotes that an elevated H/W ratio engenders heightened air temperatures, attributable to constrained air circulation and the influence of surface temperatures.

Canyon investigations elucidated the intricate relationship between enhanced ventilation dynamics and spatio-temporal aspects of 3D-UFO planning. Increased ventilation efficiency notably curtailed retention times, in contrast to scenarios with decreased efficiency. Elevated H/W ratios correlated with prolonged retention, especially on the wind-facing sides of tall buildings, in contrast to shorter retention on leeward sides in canyons. These results aligned with prior research [74], highlighting the adverse impact of increased building spacing on pedestrian ventilation. Maintaining an H/W ratio of 1.0 proved crucial in preserving a significant amount of air age along main streets, a result further substantiated by our numerical outcomes. This study also extend the work of Du et al. [75], who observed that building height variability significantly influences urban ventilation patterns, supporting our findings that indicate the strategic placement of high-rise structures can enhance air circulation.

The collision of airflows caused a three-dimensional winding stream on the leeward side of structures and an increasing channeling stream on the main street. Consequently, adjusting the aspect ratio of the three-dimensional street canyon influenced the pattern and distribution of airflow throughout the urban canopy. By holding the H/W ratio at 1.0 or less (H/W ≤ 1.0), increasing the street aspect ratio decreased ASR on the main street while elevating it within the street canyon. According to Chen et al. [76], canyons with lower H/W ratios showed increased net longwave radiation emission and enhanced convective cooling.

Maintaining H/W ≥ 1.0, an increase in the street aspect ratio elevated ASR on the main street and reduced it in the street canyon. The significance of geometry remained evident in wind performance, exemplified by mitigating heat dissipation through an increase in H/W ratio to 5.5 [77]. As the street aspect ratio increased, the surface layer's roughness in the urban environment augmented, intensifying turbulent flow and enhancing channeling flow within the main street. Notably, the tallest buildings on the leeward side experienced subdued winds. Maintaining H/W ≤ 1.0 resulted in a progressive growth of ASR value in the outermost street canyon along the flow direction. This pattern became unobservable when the H/W ratio exceeded 1.0, possibly because the street corner vortex improves vertical flux and enhances airflow in the street canyon simultaneously in building configurations with low H/W ratios. Echoing Huang et al. [78], this study emphasizes the critical importance of integrated design strategies and urban form optimization for enhancing air quality.

Through 3D-UFO can profoundly alter incoming wind patterns within urban blocks, inducing discernible shifts in turbulence characteristics and momentum flux by strategically manipulating the urban skyline. Thus, variations in urban skylines, characterized by low-rise, mid-rise, and high-rise buildings in classified sites, can either reduce or enhance urban ventilation capacity. The geometry of building arrays can be a primary factor influencing pollutant dispersion in urban blocks, demonstrating a strong correlation between density and configuration, consistent with insights from Antoszewski et al. [34].

To summarize, our findings significantly advance the field of urban planning and environmental science by providing robust, quantitative insights into the dynamics of urban airflow and pollutant dispersion. By systematically analyzing various urban block configurations using the 3D-UFO approach, this study offers valuable guidelines for designing urban environments that prioritize air quality and climate resilience. The evidence supporting the efficacy of specific building arrangements and configurations in enhancing air circulation and reducing pollutant concentrations can inform future urban planning and policy decisions, contributing to healthier and more sustainable cities.

Despite the comprehensive nature of this study, certain limitations must be acknowledged. The simulations were based on idealized urban block configurations and may not fully capture the complexities of real-world urban environments. Additionally, the focus on specific building typologies and configurations may limit the generalizability of the findings to other urban contexts. Future research should aim to incorporate more diverse urban forms and account for additional environmental variables, such as varying climatic conditions and pollutant sources. By addressing these limitations, future studies can build on our findings and develop more refined approaches to urban airflow and pollutant dispersion analysis, ultimately contributing to more effective urban design strategies.

## 6 Conclusion

This research has advanced the field of urban environmental studies by introducing the 3D Urban Form Optimization (3D-UFO) methodology, which seamlessly integrates GIS-based Land-Use and Land-Cover Change (LULCC) analysis with Computational Fluid Dynamics (CFD) simulations. Our comprehensive findings emphasize the pivotal role of urban block design in modulating air-circulation efficiency and pollutant dispersion within densely constructed environments. The study key findings are as follows.

• **Significant Variability in Air-Circulation Efficiency:** Significant variability in air-circulation efficiency was observed at both street and building levels across the evaluated urban configurations using the 3D-UFO approach.
• **Enhanced Airspeed-Ratio (ASR) and Average-Age-of-Local-Air (ALA):** Adjustments in street-aspect-ratio and building-height-ratio significantly enhanced ASR and ALA, yielding marked improvements in urban air quality metrics.
• **Optimization in Large Sites:** Large sites (approximately 4000 m^2^) demonstrated a focus on optimizing ventilation at the building level, though they often exhibited limited street-level air circulation performance.
• **Performance of High-Rise Buildings:** In larger urban settings, high-rise buildings with Height-to-Width (H/W) ratios >5.5 show a 218.5 % increase in ventilation efficiency. This enhancement, particularly notable with cubic forms and medium-density layouts, significantly enhances wind speeds, emphasizing the critical role of site characteristics in optimizing urban airflow dynamics.
• **Impact of Service Paths:** The introduction of additional service paths in small and medium-sized sites (approximately 500–2000 m^2^) enhanced street-level ventilation but frequently compromised building-level air circulation.
• **Effectiveness in Low-Rise Building Sites:** Low-rise building sites with smaller floor plans and elevated structures along block axes achieved superior indoor air circulation and building-level ventilation.
• **Challenges in Mid-Rise Building Sites:** Sites with mid-rise buildings and deep canyons (H/W ≥ 2) exhibited poor ventilation at both street and building levels. This corroborates prior research on the negative impact of high H/W ratios on air circulation, highlighting that the beneficial effects of higher H/W ratios on ventilation are context-dependent and vary based on urban configuration and building forms.
• **Maintaining Street Aspect Ratio:** Maintaining a street aspect ratio with H/W ≤ 1.0 significantly improved air circulation within the street canyon, enhancing convective cooling and reducing air retention times.

Despite these advancements, the study has limitations. The reliance on static configurations of predefined urban layouts potentially overlooks dynamic urban planning scenarios that might influence airflow dynamics differently. Furthermore, necessary simplifications in CFD simulations for computational feasibility could have constrained the accuracy of airflow predictions under specific environmental conditions. Future research should explore the socio-economic determinants shaping urban planning decisions and their implications for implementing 3D-UFO strategies. Enhanced methodologies could incorporate real-time data for dynamic modeling of urban airflow and pollutant dispersion, thereby improving predictive accuracy and robustness. Advanced computational techniques should be explored to simulate complex urban environments with greater fidelity and detail. Overall, the 3D-UFO methodology, beyond its specific applications in the current case study, holds significant promise for broader implementation in urban contexts globally. By enhancing our understanding of how urban form influences environmental quality and public health, this research contributes substantively to the evolving field of sustainable urban planning and design.

## Funding

This article is supported by the Shanghai Science and Technology Committee (Grant No. 21DZ1204500), 10.13039/501100012166National Key R&D Program of China (2023YFC3806900), 10.13039/501100012166National Key R&D Program of China (2022YFE0141400), 10.13039/501100001809National Natural Science Foundation of China (Grant No. U1913603), the Shanghai Municipal Science and Technology Major Project (2021SHZDZX0100), the Fundamental Research Funds for the Central Universities, the Postdoctoral Fellowship Program of CPSF (GZC20241226), and Special Funding for Postdoctoral Researchers from the 10.13039/501100002858China Postdoctoral Science Foundation (2024T170669).

## Data availability

All of the data are available in the manuscripts.

## CRediT authorship contribution statement

**Mehdi Makvandi:** Writing – review & editing, Writing – original draft, Visualization, Validation, Software, Project administration, Methodology, Investigation, Formal analysis, Data curation, Conceptualization. **Philip F. Yuan:** Supervision, Funding acquisition. **Qunfeng Ji:** Formal analysis. **Chuancheng Li:** Resources. **Mohamed Elsadek:** Methodology. **Wenjing Li:** Visualization, Formal analysis. **Ahmad Hassan:** Software. **Yu Li:** Visualization, Formal analysis.

## Declaration of competing interest

The authors declare that they have no known competing financial interests or personal relationships that could have appeared to influence the work reported in this paper.
