# Supplementary material for: Resilience to climate change by improving air circulation efficiency and pollutant dispersion in cities: A 3D-UFO approach to urban block design
Source: Heliyon. 2024 Aug 30;10(17):e36904. doi: 10.1016/j.heliyon.2024.e36904 (PMC11407947; doi:10.1016/j.heliyon.2024.e36904)
Supplement: Multimedia component 1 [file mmc1.docx]

**
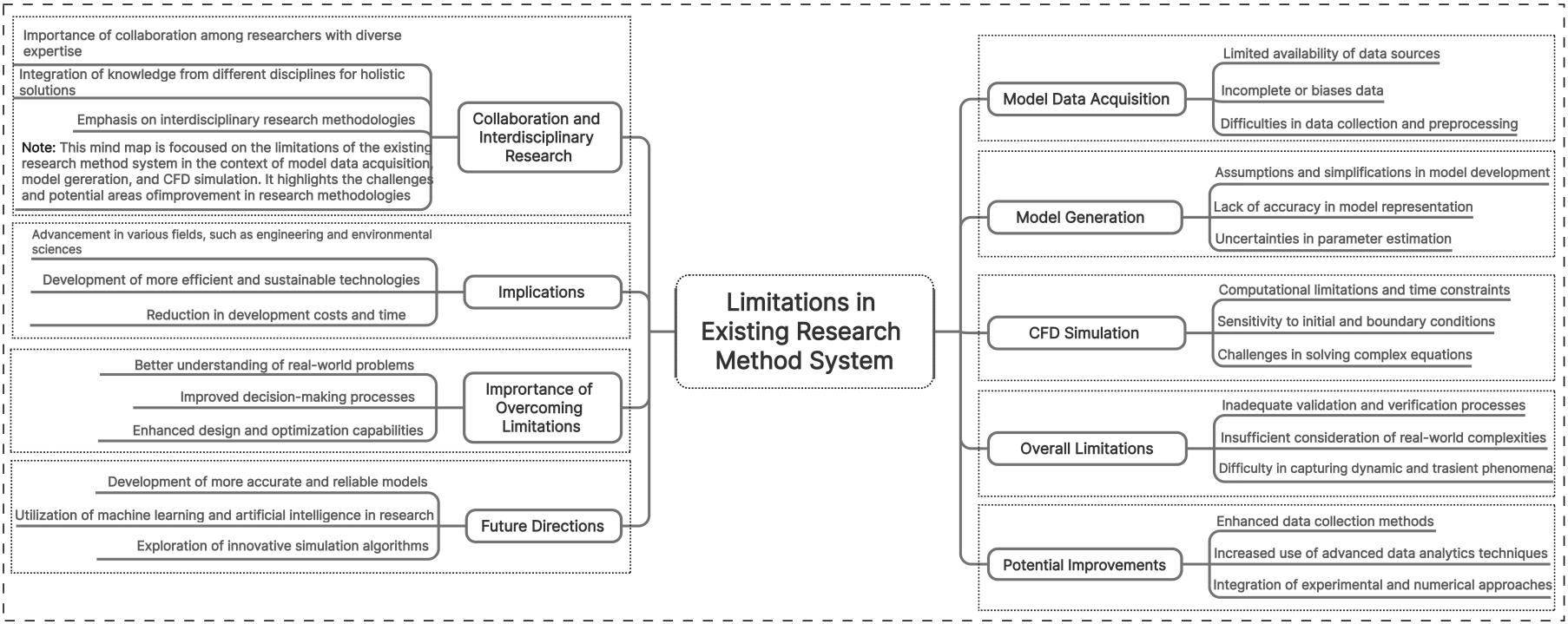
**

**Fig. S1.** Limitations of existing research method system.

| 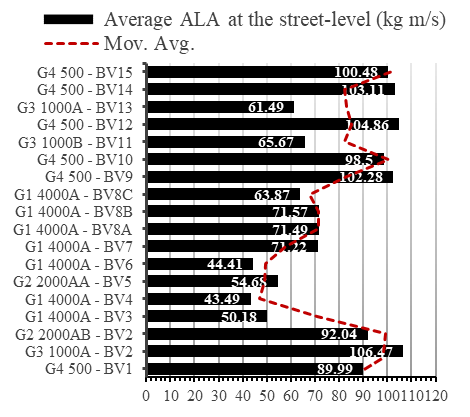(a) | 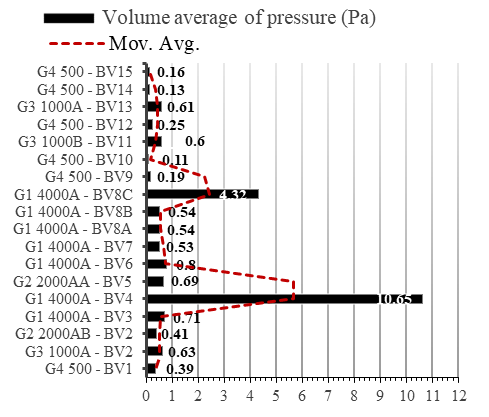 (b) | 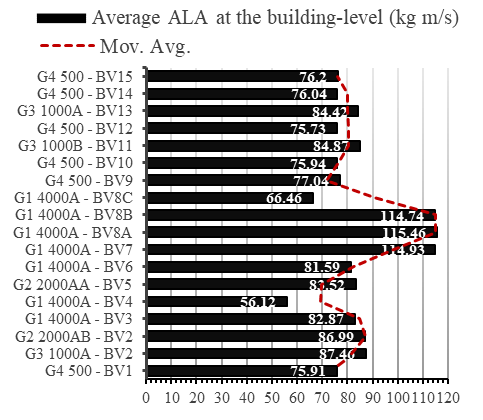 (c) | Subfigure A |
| --- | --- | --- | --- |
| 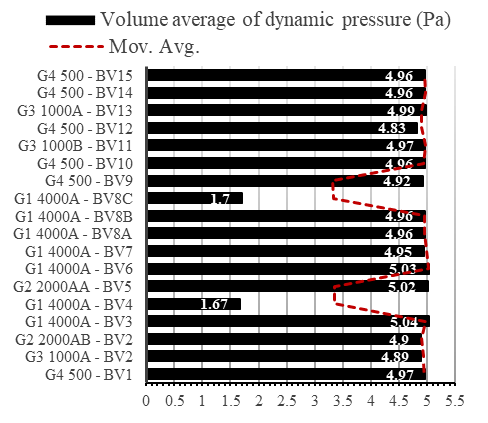(d) | (e) (f) 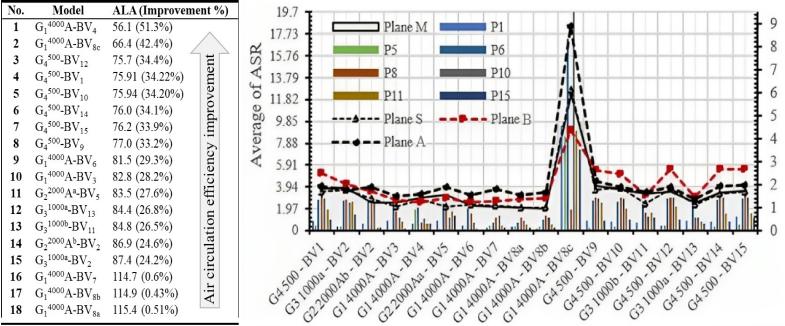 | |  |
| 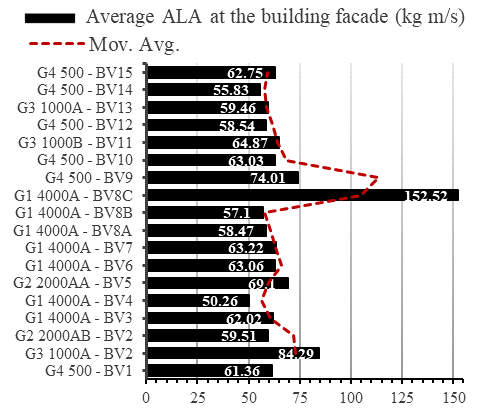(g) | 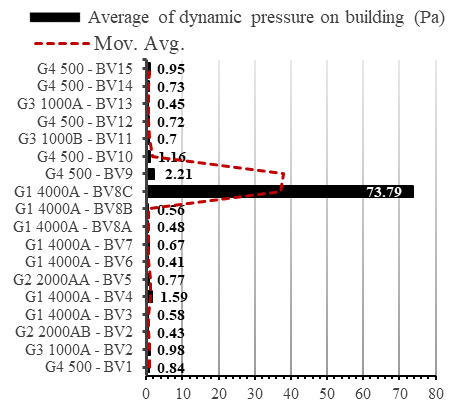 (h) | 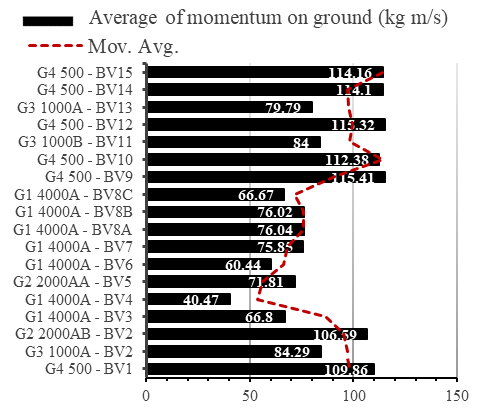 (i) | Subfigure B |
| 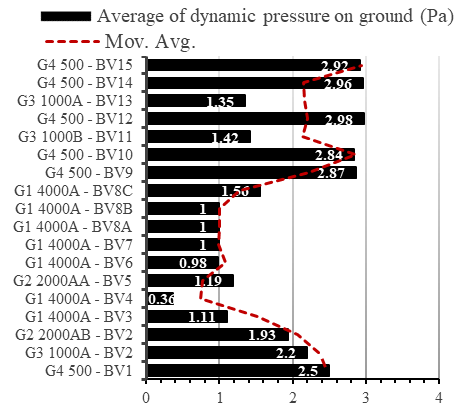(j) | 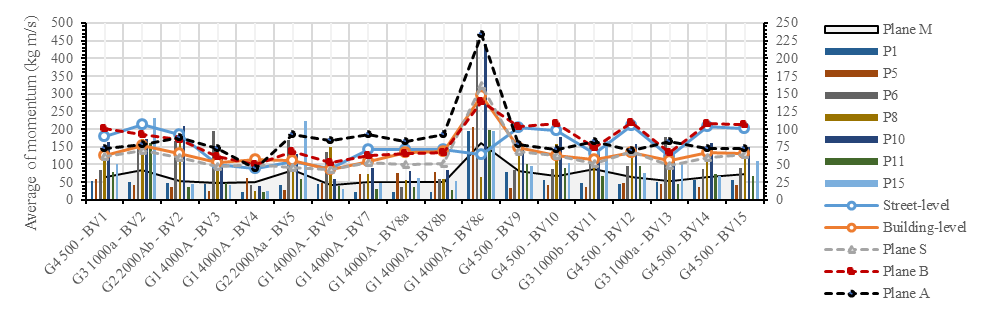 (k) | |  |

**Fig. 9.** Subfigure A encompasses (a) Average ALA at the street-level (kg m/s), (b) Volume average of pressure (Pa), (c) Average ALA at the building-level (kg m/s), (d) Volume average of dynamic pressure (Pa), (e) Performance improvement of models, and (f) Average ASR in specified points and planes at the building-level (m/s). Subfigure B comprises (g) Average ALA at the building facade (kg m/s), (h) Average of dynamic pressure on building (Pa), (i) Average of momentum on the ground (kg m/s), (j) Average of dynamic pressure on ground (Pa), and (k) Average of momentum in specified points and planes at the building-level (m/s).

Spatial distribution and statistical metrics of airflow and pressure parameters. Subfigure A encompasses (a) Street-level Average Airflow (ALA) (kg m/s), (b) Pressure Volume Averages (Pa), (c) Building-level Average ALA (kg m/s), (d) Dynamic Pressure Volume Averages (Pa), (e) Model Performance Enhancement, and (f) Building-level Average Airspeed Rate (ASR) in designated areas (m/s). Subfigure B comprises (g) Building Facade ALA (kg m/s), (h) Building Dynamic Pressure Averages (Pa), (i) Ground Momentum Averages (kg m/s), (j) Ground Dynamic Pressure Averages (Pa), and (k) Building-level Momentum Averages in specified zones (m/s).

**Fig. 9.** (a) Average ALA at the street-level (kg m/s); (b) Volume average of pressure (Pa); (c) Average ALA at the building-level (kg m/s); (d) Volume average of dynamic pressure (Pa); (e) Performance improvement of models; (f) Average ASR in specified points and planes at the building-level (m/s). (g) Average ALA at the building facade (kg m/s); (h) Average of dynamic pressure on building (Pa); (i) Average of momentum on the ground (kg m/s); (j) Average of dynamic pressure on ground (Pa); (k) Average of momentum in specified points and planes at the building-level (m/s).
